# Supplementary material for: Historical Account of Managing Overabundant Wild Asian Elephants in Myanmar by the Kheddah System of Capture: Philosophy, Principles and Practices
Source: Animals (Basel). 2024 Aug 29;14(17):2506. doi: 10.3390/ani14172506 (PMC11393919; doi:10.3390/ani14172506)
Supplement: Supplementary file 1 [file animals-14-02506-s001.zip › animals-3115480-Table supplement.pdf]

Table S1: **W**ild-caught elephants captured by decade and by capture method, from before 1960s to the end of 2000. All captures were made under the direction of MTE, the Ministry of Forestry, the Government of the Union of Myanmar.

Year capture	Method capture			
	Immobilization	Milashikar	Kheddah	Total capture
Before 1960	0	0	235	235
1960–70	49	27	508	584
1970–80	114	224	483	821
1980–90	316	8	41	365
1990–2000	153	0	3	156
Total	632	259	1270	2161
